# From supernatants to cytokines: a personal view on the early history of IL-1, IL-1Ra, TNF and its inhibitor in rheumatology

**DOI:** 10.1186/s13075-018-1607-y

**Published:** 2018-05-30

**Authors:** Jean-Michel Dayer

**Affiliations:** Faculty of Medicine, University of Geneva, Geneva, Switzerland

**Keywords:** Rheumatoid arthritis, Auto-inflammatory diseases, Collagenase, Mononuclear cell factor, IL-1, IL-1Ra, Tumour necrosis factor, TNF inhibitors

## Abstract

Long-term cell cultures developed early in the twentieth century allowed identification in their supernatants of biological mediators subsequently defined as migration factors, interferons, lymphokines, monokines, cytokines and interleukins. In rheumatology, early in the 1930s, synovial cell cultures revealed two major distinct populations, i.e. synovial fibroblasts and monocyte-macrophages. Discovery of the interstitial collagenase (MMP-1) and its role in tissue destruction, such as in rheumatoid arthritis (RA), raised the question of the cellular source for this enzyme. My personal interest in the field was driven by the lack of understanding for the link between tissue destruction and immunology. This triggered our seminal contribution to the field, establishing in 1976–79 at the Arthritis Unit (Massachusetts General Hospital, with SM Krane) that a mononuclear factor (MCF, around 15 kDa) produced by stimulated macrophage, under direct contact with activated T cells, induced large amounts of collagenase and prostaglandin E_2_ (PGE_2_, a bone resorbing agent) in human synovial fibroblasts from RA patients. Our original “MCF” biological observations preceded cloning, and recombinant IL-1β confirmed the biological activity of the purified natural IL-1. Following my return to Geneva in 1980 and searching for a high level of IL-1 in urine and serum of patients with high fever or Still’s disease, to our surprise—“a finding of absence”—we found that IL-1 was masked by a factor of approximately 17 kDa and first presented this in 1984 at the Fourth International Lymphokine Workshop. In 1987, before IL-Ra cloning, my co-worker P Seckinger and I demonstrated first-time observation in cytokine biology that the mechanism was due to the inhibition of IL-1 binding the cell surface receptor, leading to the concept of IL-1 receptor antagonist (IL-1Ra). Having reported in 1985 that TNF/cachectin also induced collagenase and PGE_2_ in human synovial cells, we found that IL-1Ra did not block TNF-α but was due to another inhibitor. As other investigators, we confirmed that this inhibitory factor was a soluble TNF receptor. The years between the 1970s and 1990s were probably the most exciting period in the field of cytokines and cytokine antagonists; it gave rise to two concepts in the cytokine field—one of the receptor antagonist, and the other of soluble receptor antagonists.

The concept of supernatant in cellular biology originated in 1885 when Wilhelm Roux set up a tissue culture to maintain a medullary plate of an embryonic chicken in a warm saline solution for several days. Ross Harrison developed the methodology of cell culture (in 1907) that consisted of the “hanging drop technique”—whereby a small piece of tissue was placed in a drop of medium (including serum)—allowing cells to migrate from tissue to the surrounding environment. Further landmarks were set first in 1911 by Alexis Carrel and Montrose Burrows with their long-term aseptic technique of culture tissue cells and its possibilities, and later in 1935 by Alexis Carrel and Charles Lindbergh who devised new cell and organ culture systems. In rheumatology, a seminal description of “the form and function of synovial cells in tissue cultures” by Ernst Vaubel at the Rockefeller Institute Hospital (in 1933) highlighted early on the difference between macrophage-like cells and fibroblast-like cells, introducing the terminology of “synovioblast”, which in turn gave rise to the question of differentiation from one to the other [1].

The history of the precursor of “cytokines” dates to allergy and infection by permeability factors from supernatant of tissue sensitized to tuberculin by Hans Zinsser and Takeo Tamiya (in 1926) and the conceptualization of its mechanisms by Arnold Rich (in 1927). In 1958, Byron Waksman and Margit Matolfsky realized that “sensitized” macrophages were stimulated rather than damaged by tuberculin protein. In 1957, Alick Isaacs and Jean Lindenmann coined the term “interferon” when observing that the inhibition (“interference”) of the growth of live influenza virus in chicken embryo chorioallantoic membranes was mediated by a protein released by cell membranes treated with heat-inactivated influenza virus. In 1966, a substance inhibiting the migration of normal peritoneal exudate macrophages was reported simultaneously by John David as well as by Barry Bloom and Boyce Bennett, termed migration-inhibitory factor (MIF). Nancy Ruddle and Byron Waksman as well as William Kolb and Gale Granger described lymphotoxin (LT) in 1968, later also referred to as TNF-β. The terminology of “lymphokine, monokine” was employed by Dudley Dumonde in 1969, “lymphokines” being defined as “non-antibody mediators of cellular immunity generated by lymphocyte activation”, pointing to the implication of certain lymphocytes in the innate immune system. The “lymphocyte-activating factor” (LAF) was identified by Igal Gery and Byron Waksman in 1972, as was the osteoclast-activating factor (OAF) by John Horton and Lawrence (Larry) Raisz in the same year. In 1974, Stanley Cohen introduced the term “cytokine” as a non-leukocyte also producing cytokines and pointed out that such molecules could be produced by many cells in the body. That same year, Charles Dinarello, Nathan Goldin and Sheldon Wolff described human “endogenous leucocyte pyrogen” (EP).

In rheumatology, from the 1950s until the 1970s, two different approaches in the pathogenesis of rheumatoid arthritis (RA) were concomitantly debated: one focusing on the role of the cellular and humoral immune components, including the immune complexes; the other on the biochemistry of structural matrix components of tissue, mainly collagens and proteoglycans. The terminology of “connective tissue diseases” was in fashion, but the link between immunology and the extracellular matrix (ECM) was poorly understood. Research on the ECM structures prompted biochemists to unravel structural and genetic anomalies of the matrix, leading to the concept that abnormal or denatured endogenous proteins, or collagen peptides—being recognized as foreign antigens—gave rise to autoimmune diseases such as RA. Subsequently, during the 1970s and 1980s, progress in enzymology encouraged scientists to investigate aberrations in enzymes and proenzymes, as well as their autoactivation, suggesting that part of the pathogenesis of RA could be linked to enzyme abnormalities. In the early 1970s, investigators backtracked to pay closer attention to cellular aspects of the synovial tissue of RA patients (pannus), but very little was known at that time about the molecular pathways between immune cells and mesenchymal cells.

At the end of my specialization in internal medicine in Geneva, I was intrigued by the lack of connection between the field of “connective tissue diseases” involving matrix, tissue destruction, fibrosis, collagen and proteases and the field of “connectivity” involving immune cells, inflammation and mediators. The head of the Department of Medicine, Alex F. Müller, in Geneva advised me in 1974 to spend some time (actually 7 years!) at Massachusetts General Hospital (MGH), Boston, MA, USA. At MGH, Jeremy Gross and Charles Lapière of the Robert W. Lovett Memorial Group for the Study of Diseases Causing Deformities had in 1962 discovered interstitial collagenase (now termed MMP-1) released during limb regeneration in amphibia (tadpole tissue) [2]. This led the same group—the Arthritis Unit headed by Stephen Krane—to the discovery of human collagenase from rheumatoid synovium in organ culture [3].

On my arrival at the Arthritis Unit of MGH, Stephen Krane assigned me the project of isolating the RA synovial tissue cells responsible for producing collagenase. At the time, macrophages were thought to be the main source of proteases, fibroblasts being the main source of ECM. After a year of vain and frustrating efforts to detect collagenase in isolated human macrophages from synovial tissue, it was finally in 1976 that I identified large amounts of collagenase and prostaglandin E_2_ (PGE_2_) produced in primary mixed cultures of synovial fibroblast cells (adherent stellate fibroblast-like cells); PGE_2_ being considered at the time to be an important bone-resorbing agent [4]. However, although collagenase production decreased progressively, it was still present when the mononuclear cells had disappeared after cell culture passages, suggesting that the previous contact of the synovial fibroblasts with the mononuclear cells was memorized. To unravel this phenomenon, blood mononuclear cells were isolated, stimulated and added to the late-passage fibroblast cultures, resulting in the reactivation of large amounts of collagenase and PGE_2_ production, along with the stellate aspect of fibroblast-like cells (Fig. 1). It turned out that this phenomenon was mediated by a partially purified factor of around 15 kDa that we termed “mononuclear cell factor” (MCF) [5, 6]. Consequently, synovial-like fibroblasts were the main source of interstitial collagenase and PGE_2,_ but only in the presence of mononuclear cells. Indeed, interestingly, the cells producing most of the collagenase were also the principal source of their substrate, collagen. Based on this observation in 1979, when analysing the respective functions of the subpopulations of the mononuclear cells, we demonstrated that T lymphocytes (TL) stimulated monocyte-macrophages (MΦ) to produce MCF, thus establishing the sequential pathways from TL to MΦ and to synovial fibroblast activation. Except for the absence of B cells, the proposed scheme is still valid after 40 years [7, 8] (Fig. 2). Subsequently, we found that the direct contact between TL and MΦ was essential and that it depended on the cell-surface molecules [9]. With regard to RA, self-associating IgG rheumatoid factors proved to stimulate directly MCF production by monocytes [10].

**Fig. 1:**
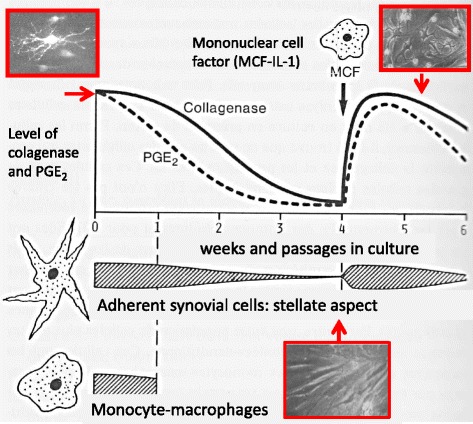
Reactivation by MCF of large amounts of collagenase and PGE_2_ production, along with the stellate aspect of fibroblast-like cells [5, 6]. IL interleukin, PGE_2_ prostaglandin E_2_

**Fig. 2:**
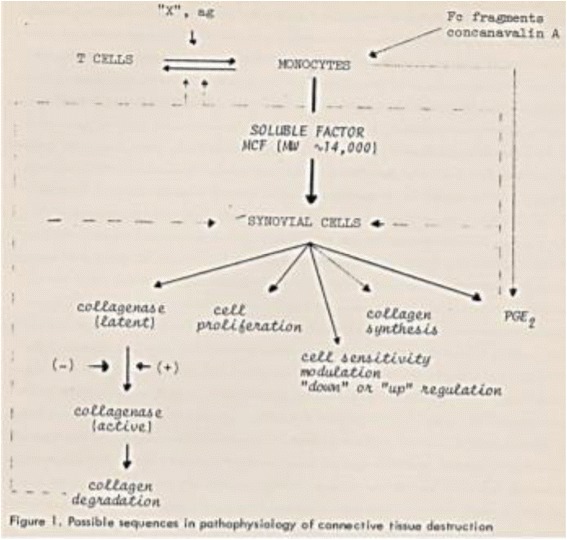
Sequential pathways from TL to MF and to synovial fibroblast activation [7]. MCF mononuclear cell factor, MΦ monocyte-macrophages, PGE_2_ prostaglandin E_2_, Fc Fragment crystallizable of immunoglobulin, ag antigen

On purification, our “MCF” exhibited similar properties to LAF with a molecular weight of about 15 kDa and shared chromatographic and other biochemical properties [11]. It was not until 1979 that the nomenclature of IL-1 was coined at the Second International Lymphokine Workshop in Ermatingen, Switzerland, in a letter to the Editor of the *Journal of Immunology* entitled “Revised Nomenclature for Antigen-Nonspecific T Cell Proliferation and Helper Factors” [12], thus unifying previous factors with similar biomedical and biologic properties to LAF such as MCF. The monocyte products were defined as interleukin-1 and the T-cell products as interleukin-2; both were unrestricted to H-2 activity. In contrast to IL-2, which is essential for maintaining long-term T-cell cultures, IL-1 was unenabled [12]. Using cartilage organ culture as a target, John Dingle and Jeremy Saklatvala isolated a cartilage catabolic factor from synovium in 1979, later in 1986 called “Catabolin”. The precursor of TNF-α, cachectin/TNF, was identified in 1985 as a lipoprotein lipase-suppressing hormone secreted by endotoxin-induced cells in Anthony (Tony) Cerami’s laboratory.

Then followed my return to Geneva in 1980, when several research groups were competing for the same objective: to clone IL-1. Starting with lectin-stimulated human blood mononuclear cells, we isolated poly(A) RNA and studied its translation after microinjection into *Xenopus laevis* oocytes. The mRNA translation products stimulated collagenase and PGE_2_ production in human rheumatoid synovial cells and dermal fibroblasts. The size of MCF mRNA was estimated at 10 S [13]. Our original biological observations preceded the cloning, the cDNA of IL-1β being reported by Charles Dinarello in 1984, and by means of recombinant IL-1β we confirmed the biological activity of our purified natural IL-1 [14].

At that same time, we needed large amounts of biological material to purify IL-1. It therefore occurred to us that patients who had great numbers of monocytes, such as patients with monocytic leukaemia (M5) or high fever, would be an ideal source. However, to our surprise—“a finding of absence”—no IL-1 biological activity could be detected in urine and serum of such patients or others with high fever such as those suffering from adult Still’s disease. Puzzled by this observation, we reasoned that the biological activity had to be present, while being masked. In fact, IL-1 was indeed masked by a factor of approximately 17 kDa. This seminal observation was first presented in 1984 at the Fourth International Lymphokine Workshop [15] and published in full papers [16, 17]. By coincidence, at that intense meeting, the first sequence of IL-1β was presented by Charles Dinarello. Afterwards, for us the most important goal was to unravel the mechanism of inhibition. Inspired by the endocrinology field during my stay at MGH, myself, my co-worker Philippe Seckinger and colleagues in Lausanne succeeded in reaching that goal. Indeed, by using the binding to EL4-6.1 cells we demonstrated in 1989 that a purified ^125^Iodinated natural inhibitor (apparent m.w. around 17 kDa) competed with natural IL-1, which confirmed for the first time the concept of interleukin-1 receptor antagonist (IL-1Ra), and we consequently described how a natural cytokine antagonist blocked the binding of another cytokine, and this from the same family [18]. The principle of the competitive binding assay of the IL-1 inhibitor to IL-1 at the IL-1 receptor level was crucial for researchers at Synergen at Boulder, Colorado, USA who purified and cloned IL-1Ra in 1990. We then collected more than 100 L of urine from patients to purify to homogeneity the IL-1 inhibitory factor [19]. The clinical role of IL-Ra was established in 1987 in a clinical observation when analysing the serum and urine of children with systemic juvenile chronic arthritis (JRA), a typical inflammatory syndrome. An increase in IL-1 inhibition (IL-Ra) was observed following the peak of the fever [20] (Fig. 3, adapted scheme).

**Fig. 3:**
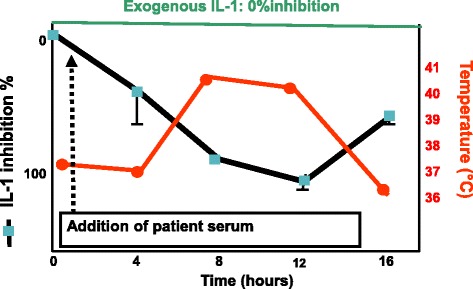
IL-1 inhibition (bioassay in presence of constant amounts of exogenous IL-1) following the peak of the fever induced by serum from children with systemic juvenile chronic arthritis (adapted scheme from [20]). IL interleukin

The events involving the transition from IL-1-Ra to TNF inhibitor occurred naturally. In the middle of the 1980s, overlapping biological activities between IL-1 and tumour necrosis factor (TNF-α) were under discussion. Having reported in 1985 that TNF-α also induced collagenase and PGE_2_ in human synovial cells, we found that the urinary inhibitor of IL-1 activity—which affected both interleukin-1α and IL-1β—did not affect TNF-α [21, 22]. However, when analysing other chromatographic fractions, we isolated and purified a human inhibitor to TNF-α [23]. As did other investigators, we confirmed that this factor was the soluble TNF receptor [24].

Further work in our laboratory was focused on the effect of direct cell–cell contact on the regulation of IL-1, TNF and their respective inhibitors such as IL-1Ra and soluble TNF receptors (review [25, 26]).

The years between the 1970s and the 1990s were probably the most exciting period in the field of cytokines and cytokine antagonists; it gave rise to two concepts in this field—one of the receptor antagonist, and the other of soluble receptor antagonists.

Cloning of cDNA of IFN-α and IFN-β in 1980 and of cDNA of TL (TNF-β) and TNF-α and IL-1 in 1984 was achieved, followed by that of IL-1Ra in 1990. This has revolutionized the field, and since the 1990s recombinant molecules and specific monoclonal antibodies have allowed the expansion of the field of signal transduction, the dissection of the hierarchy of the different cytokines depending on the types of disease and therapeutic approaches. The latest interleukin is now IL-40 specifically produced by B cells.

Still, discussion as to the cells responsible for the onset of RA is still the subject of debate and the initial role of synovial fibroblasts continues to be of interest, since there is clear evidence that their subpopulations express different genes and respond differently to cytokines [27].

Even if IL-1 blockade is marginally successful in RA, IL-1Ra—and probably other antagonists to the IL-1 family—has become most important in the treatment of several auto-inflammatory diseases, reminding us of our seminal observation of the inhibitor in JRA [20], whilst TNF or IL-6 has been the key cytokine to be blocked in RA and several other chronic inflammatory diseases.

## Abbreviations

ECM: Extracellular matrix
EP: Endogenous leucocyte pyrogen
IgG: Immunoglobulin G
IL: Interleukin
JRA: Juvenile chronic arthritis
LAF: Lymphocyte-activating factor
LT: Lymphotoxin
MCF: Mononuclear cell factor
MGH: Massachusetts General Hospital
MIF: Migration-inhibitory factor
mRNA: Messenger ribonucleic acid
MΦ: Monocyte-macrophages
OAF: Osteoclast-activating factor
PGE_2_: Prostaglandin E_2_
RA: Rheumatoid arthritis
TL: T lymphocytes
TNF: Tumour necrosis factor
